# The Role of Subjective Vitality Among Older Adults from a Self-Determination Theory Perspective: A Scoping Review

**DOI:** 10.3390/geriatrics11040091

**Published:** 2026-07-20

**Authors:** James Dawe, Christina M. Frederick, Sara Manganelli, Elisa Cavicchiolo, Fabio Alivernini, Giulia Raimondi

**Affiliations:** 1 Department of Humanities and Social Sciences, Mercatorum University, Piazza Mattei 10, 00186 Rome, Italy; 2 Department of Behavioral and Social Sciences, Embry-Riddle Aeronautical University, Daytona Beach, FL 32144, USA; frederic@erau.edu; 3 Department of Developmental and Social Psychology, Sapienza University of Rome, Via dei Marsi 78, 00185 Rome, Italy; sara.manganelli@uniroma1.it (S.M.);; 4 Department of Systems Medicine, Tor Vergata University of Rome, Via Montpellier 1, 00133 Rome, Italy; elisa.cavicchiolo@uniroma2.it; 5 BeSSA Department, Wellbeing, Health and Environmental Sustainability, Sapienza University of Rome, Piazzale Aldo Moro 5, 00185 Rome, Italy

**Keywords:** subjective vitality, older adults, scoping review, SDT

## Abstract

**Background:** Aging involves several social, psychological, and biological changes, including changes in perceived energy. Within Self-Determination Theory, Subjective Vitality refers to the experience of having energy available to the self. Despite its relevance for older adults, no review has mapped how Subjective Vitality has been studied in this population. This scoping review aimed to map the literature on Subjective Vitality in older adults and identify gaps in the evidence base. **Method:** Following Joanna Briggs Institute guidance, five electronic databases were searched for peer-reviewed quantitative articles published between 1997 and 2026 in which Subjective Vitality was assessed in samples with a mean age of 65 years or older. Of the 1915 records identified, 11 reports were included, corresponding to eight independent samples. **Results:** Studies were conducted in a limited number of countries, predominantly Western, and showed substantial heterogeneity across research domains. Subjective Vitality was most often assessed using the Subjective Vitality Scale, although different versions and response formats were used, and psychometric or adaptation evidence specific to older adults was limited. Potential antecedents and mechanisms included basic psychological need satisfaction, autonomy support, autonomous regulation, intrinsic goals, flow, social contacts, ego integrity, and meaning in life. Intervention studies suggested that leisure education and endurance training may support Subjective Vitality, although evidence remains limited. **Conclusions:** Future studies should use clearer sampling procedures, validated and well-reported measures, and stronger longitudinal and intervention designs capable of testing temporal and causal relationships.

## 1. Introduction

People aged 65 years and older represent a rapidly growing segment of the global population. The United Nations estimates that their number will more than double, rising from 761 million in 2021 to 1.6 billion in 2050 [1]. Population aging is particularly advanced in developed countries, where people aged 65 years and older represented approximately 20% of the population in 2023 [2]. This demographic shift represents one of the major challenges that societies will face in relation to health, care, financial support, societal participation, and quality of life.

Later life is marked by a complex set of changes that involve social, physical, and psychological aspects across several areas of life. For example, for most individuals, entering this stage of life may coincide with retirement, creating the opportunity for new forms of engagement, including spending more time with family, returning to education, taking on new occupational roles, or resuming interests that had been postponed over the years [3,4]. However, these opportunities unfold alongside age-related biological changes, such as increased chronic illness and functional decline, both of which can negatively affect quality of life [5,6]. In this scenario, feeling less energetic and more fatigued is a common experience among older adults [7], particularly evident when contrasted with earlier life stages [8]. This reduction in energy may impact everyday functioning, influencing the capacity to engage in daily activities, maintain social participation, and preserve life satisfaction [7,9,10,11]. Reduced energy may also lead to a more sedentary lifestyle and poorer health outcomes [12]. Indeed, the literature suggests that older adults’ quality of life is associated with social factors, health, physical activity, and independence in activities of daily living [13,14,15,16,17], all areas that could be affected by energy reduction.

Within Self-Determination Theory (SDT), Ryan and Frederick [10] developed the concept of Subjective Vitality, defined as “a positive feeling of aliveness and energy available to the self” (p. 529). Subjective Vitality has been considered an important indicator of an individual’s well-being, flourishing, and maturation, and is associated with better psychological and physical health [18,19]. In contrast, its depletion has been associated with poor health and ill-being [18,19]. At the same time, it can serve as an energetic resource for older adults that can be increased, decreased, and mobilized to manage daily tasks and adapt to change and new challenges that characterize this phase of life. Thus, it is important to identify the psychological and contextual factors that may support or undermine it.

According to the SDT framework [18,19], Subjective Vitality is affected by the satisfaction (or frustration) of three basic psychological needs: autonomy (i.e., a feeling of volition and acting with choice), competence (i.e., a feeling of effectiveness and of mastering a task), and relatedness (i.e., a feeling of connection with significant others and of belonging). Satisfying these needs provides the energy that fuels action [20], and they are considered principal antecedents of subjective vitality. Following this theory, needs may mediate between psychological and contextual factors and subjective vitality. For example, Adie et al. [21] observed that an autonomy-supportive environment can enhance subjective vitality among sport participants, and that this association was mediated by autonomy and competence satisfaction. As most of this research has been done with younger adults, it remains unclear whether this pathway has been considered in the literature on older adults, which psychological and contextual factors have been examined, and how these relationships have been modeled. Understanding this would not only help researchers develop further studies but also help the development of more successful interventions.

Before such antecedents and mechanisms can be interpreted confidently, however, it is necessary to consider how Subjective Vitality has been measured. Recent studies have examined the assessment of Subjective Vitality across different populations and developmental stages, supporting the psychometric adequacy of these measures [22,23]. Ryan and Frederick [10] originally developed the seven-item Subjective Vitality Scale (SVS), which was later examined in shorter versions, including six-item [24] and five-item forms [25]. Comparative psychometric studies have suggested that shorter versions, particularly the five-item version, may show stronger fit, reliability, and validity [25,26]. However, only limited validation evidence is available specifically for older adults, with Couto et al. [23] validating a six-item version in this population. More recently, Dawe et al. [18] validated the Subjective Vitality and Depletion Scale (SVDS), which assesses Subjective Vitality together with Subjective Depletion in older adults. Clarifying which instruments have been used and which psychometric evidence supports them is therefore important for evaluating the robustness and comparability of findings in this literature.

Despite growing interest in vitality in older age, no review has yet mapped the literature on Subjective Vitality in older adults specifically from an SDT perspective, including how the construct has been conceptualized, measured, and linked to SDT-related, as well as other relevant antecedents. The present scoping review aimed to map the extent and characteristics of the literature on Subjective Vitality in the older adult population and identify where there are gaps in the evidence-based literature. Specifically, this review addresses the following questions: (1) In which populations and research domains has Subjective Vitality been studied in older adults? (2) How has Subjective Vitality been measured, and what psychometric or adaptation evidence is available? (3) Which SDT-related and other antecedents, mechanisms, and intervention approaches have been examined in relation to Subjective Vitality in older adults? (4) What methodological gaps characterize the current literature? Mapping this literature is important for clarifying how Subjective Vitality has been studied in older adults, identifying methodological and measurement gaps, and informing future research and intervention development. Given that the primary focus of the present scoping review was on how the construct was measured and the modeled association of subjective vitality, we focused on quantitative studies.

## 2. Methods

Scoping reviews are well suited for mapping existing evidence on a topic and identifying gaps in a specific field [27]. In contrast to traditional systematic reviews, scoping reviews have a broader scope and are ideal when it is still unclear what other, more specific questions can be posed and valuably addressed by a more precise systematic review [28]. Given the absence of previous synthesis focusing on subjective vitality from an SDT perspective among older adults, a scoping review was deemed to be the most appropriate method to identify, report, and describe study characteristics. This scoping review was conducted in accordance with the Joanna Briggs Institute (JBI) guidance for scoping reviews [27,29] and the Preferred Reporting Items for Systematic Reviews and Meta-Analyses extension for Scoping Reviews (PRISMA-ScR) [30]. To ensure transparency, the PRISMA-ScR checklist [30] was filled out (File S1). This scoping review was not registered.

### 2.1. Eligibility Criteria

To define inclusion and exclusion criteria, the Population/Participants, Concept, and Context (PCC) [29] framework was applied. Specifically, the population was older adults, widely defined as individuals 65 years and above [31], with no restrictions concerning health or other characteristics. To be included in the study, all participants had to be over 65. Studies were included when they explicitly assessed Subjective Vitality using the Subjective Vitality Scale, the Subjective Vitality and Depletion Scale, or another measure explicitly grounded in SDT’s conceptualization of Subjective Vitality. No restrictions were applied regarding setting, geographical location, or substantive research domain. Only peer-reviewed, original quantitative articles published in English were considered for this review.

### 2.2. Search Procedure

The following electronic databases were selected: Scopus, Web of Science, CINAHL, PsycINFO, and MEDLINE. The databases were searched from inception to 18 March 2026. Moreover, on the same date, the SDT website (https://selfdeterminationtheory.org/research/, accessed on 18 March 2026) was searched to identify additional relevant studies, using vitality as a filter, as well as the references of included articles. The keywords used in the search referred to three main themes: older adults (e.g., over 65), subjective vitality, and the SDT theoretical framework (e.g., self-determination). Supplementary Material File S2 provides the full details on the final search string.

### 2.3. Study Selection

After identification, records were uploaded to Rayyan (Rayyan; Qatar Computing Research Institute, Doha, Qatar) [32] for duplicate removal and screening. Two authors screened the titles and abstracts of potentially eligible records. Full papers were retrieved and consulted if the abstract did not provide the necessary information to verify if they met the criteria (especially the precise age of the individuals included in an article) for inclusion in the review. Two authors retrieved and assessed the full texts for eligibility. Any disagreement between the two review authors over the eligibility of a particular study was resolved through discussion with a third review author.

### 2.4. Data Extraction

A pre-defined Microsoft Excel (version 2606; Microsoft Corporation, Redmond, WA, USA) chart was prepared for data extraction. Information from the articles was extracted by the first author (data concealed for blind peer-review). The chart included the following information: (1) citation and year of publication; (2) sample characteristics (i.e., country, N, mean and standard deviation of the age, % of females); (3) Population; (4) research domains; (5) study design; (6) measure of subjective vitality and psychometric characteristics; (7) SDT antecedents (i.e., variables grounded in the SDT framework, such as basic psychological need satisfaction and autonomous motivation); (8) non-SDT antecedents (i.e., any other variables that the included studies examined as correlates, predictors, or mechanisms of Subjective Vitality). Research domains categories and non-SDT antecedents were not prespecified, but coded during the data extraction process. Data extraction was conducted by the first author and checked by a second author. Discrepancies were resolved through discussion.

### 2.5. Methodological Quality Appraisal

Even though quality appraisal is not usually conducted for scoping reviews [27], to better inform further research on the areas that still need to be improved in this field, the Mixed Methods Appraisal Tool (MMAT, version 2018; [33]) was used. This tool was chosen because it accommodates the range of quantitative designs represented in the included studies (randomized controlled trials, non-randomized intervention studies, and quantitative descriptive studies) within a single appraisal framework. The MMAT comprises 25 items, five for each study type: (1) qualitative research, (2) quantitative randomized controlled trials, (3) quantitative non-randomized studies, (4) quantitative descriptive studies, and (5) mixed-methods studies. Each study was appraised using only the five MMAT items corresponding to its specific design. Items are scored “Yes” if the criterion is met, “No” if it is not, or “Can’t tell” when information is insufficient. For each study, we calculated an overall quality score as the percentage of “Yes” responses [34]. Given that the focus was on quantitative studies, items regarding qualitative and mixed-method studies were not used. Two independent reviewers assessed the quality of the included studies. Disagreements were resolved through discussion until a consensus was reached.

### 2.6. Data Analysis and Presentation

Characteristics of the included studies were reported through the use of tables. All findings were then synthesized into tables and narrative summaries, grouped according to the four research questions. Tables were generated to describe and provide an overview of the main findings on the literature on subjective vitality among older adults.

## 3. Results

### 3.1. Study Selection

**Figure 1:**
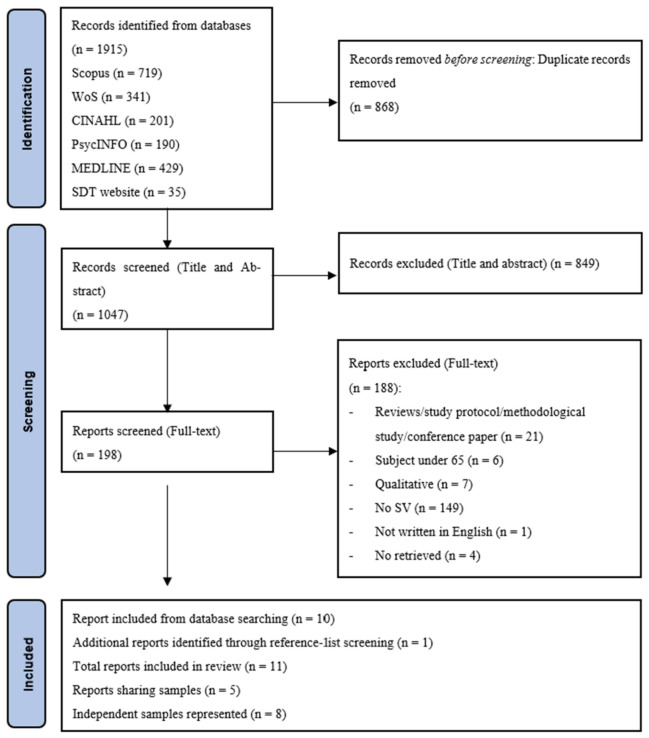
PRISMA flow diagram. Note. Counts refer to reports. The 11 included reports correspond to 8 independent samples: the three Solberg et al. [35,36,37] reports derive from one exercise-intervention project, and the Van der Kaap-Deeder et al. [38] and Vermote et al. [39] reports derive from one COVID-19 survey project.

The database search yielded 1915 records, of which 868 were duplicates. After duplicate removal, 1047 records were screened by title and abstract, and 849 were excluded. The remaining 198 reports were assessed in full text. Ten reports met the eligibility criteria and were included. One additional eligible report was identified through screening the reference lists of included articles, resulting in a total of 11 included reports. After accounting for overlapping samples, these reports corresponded to eight independent samples. Specifically, the three Solberg et al. [35,36,37] reports were treated as one exercise-intervention project, and the Van der Kaap-Deeder et al. [38] and Vermote et al. [39] reports were treated as one COVID-19 survey project. Unless otherwise specified, descriptive counts in the Results refer to these eight independent samples. Figure 1 presents the PRISMA flow diagram summarizing the screening process.

### 3.2. Studies’ Characteristics

Included reports were published between 1999 and 2026, with five reports published from 2020 onward. Across the eight independent samples, a total of 2142 participants were included, with sample sizes ranging from 42 to 726 participants. Approximately 57% of participants were female (*n* = 1229), although one sample did not report the number or percentage of female participants. Mean age ranged from 69.12 to 83 years. The independent samples were drawn from heterogeneous geographical contexts. Two were from Taiwan; the remaining samples were mainly from Europe (Norway, the United Kingdom, Belgium, and France), with one from the USA and one including participants from both Italy and the USA. Table 1 reports the main characteristics of the included reports.

Most independent samples (*n* = 9) were coded as general, meaning that they involved broadly defined older-adult populations, without specific clinical, functional, or social characteristics reported. One sample consisted of nursing home residents. The research domains in which Subjective Vitality was examined were also heterogeneous. Two independent samples were examined in the domain of leisure activities. The remaining samples were examined in the domains of physical activity, active aging, health, general validation, COVID-19 lockdown, and well-being, with one sample in each domain.

Regarding study design, most independent samples were included in observational studies. Four samples were examined in cross-sectional studies, two in randomized controlled trials (RCTs), and one in a non-randomized study with intervention (NRSI). One COVID-19 survey sample was reported across two related reports, one using cross-sectional analyses and the other using prospective analyses.

### 3.3. Measures of Subjective Vitality, Their Psychometric Properties, and Adaptations

As shown in Table 2, Subjective Vitality was assessed using a version of the Subjective Vitality Scale (SVS) in nearly all independent samples (*n* = 7), whereas the new Subjective Vitality and Depletion Scale (SVDS) was used in one sample. Different versions of the SVS were adopted. Among SVS-based studies, the number of items varied substantially, ranging from a single-item assessment to the seven-item scale. The six-item version was used in two independent samples, the five-item version in two, and the seven-item, four-item, and one-item versions in one sample each. The SVDS study used the three-item vitality subscale. Response scales also varied: four independent samples used a seven-point response scale, two used a five-point response scale, and two used a four-point response scale. Reflecting the geographical heterogeneity of the included studies, the questionnaires were administered in several languages, including English (*n* = 3), Chinese/Taiwanese Mandarin (*n* = 2), Italian (*n* = 1), Norwegian (*n* = 1), Dutch (*n* = 1), and French (*n* = 1). Because one study used two language versions, language counts exceed the number of independent samples.

Psychometric reporting was uneven. Internal consistency coefficients were reported in five independent samples, including four using the SVS and one using the SVDS vitality subscale. Alpha values were generally in the acceptable-to-excellent range. However, reliability was not reported in some samples and was not applicable in studies using a single-item assessment. Evidence beyond internal consistency was rare. With the exception of Dawe et al. [18], which explicitly evaluated the factor structure, reliability, measurement invariance, and validity of the SVDS, most included studies relied on previously validated measures without providing new psychometric evidence for the specific version used.

Regarding adaptation to older adults, only two independent samples used versions of the SVS for which previous evidence in older populations was reported or cited. Four samples adopted versions of the SVS for which previous validation was cited, but without older-adult-specific validation or adaptation evidence reported in the study. One independent sample used a single-item assessment of Subjective Vitality, for which reliability was not applicable and validation evidence was not reported.

### 3.4. Antecedents and Mechanisms of Subjective Vitality in Older Adults

#### 3.4.1. Correlational Evidence

Of the eight independent samples, SDT theoretical antecedents or mechanisms of Subjective Vitality were examined in five (Table 3). The three basic psychological needs for autonomy, competence, and relatedness satisfaction were examined in three independent samples [38,41,44]. Overall, need satisfaction was positively associated with Subjective Vitality. However, the pattern of unique predictors differed across studies. In Chang [41] and Vanhove-Meriaux et al. [44], relatedness satisfaction was the only need that remained a significant unique predictor of Subjective Vitality, whereas autonomy and competence satisfaction were not significant. In contrast, Van der Kaap-Deeder et al. [38] found that, when the three needs were examined separately, competence satisfaction was the only need uniquely associated with Subjective Vitality, whereas autonomy and relatedness satisfaction were not. Perceived competence was examined specifically in Solberg et al. [36], showing that exercise was associated with greater perceived competence, which was then related to higher Subjective Vitality, although this mediating pathway was only marginal. Finally, need thwarting was examined only in Vanhove-Meriaux et al. [44]. Autonomy, competence, and relatedness thwarting were negatively correlated with Subjective Vitality; however, these dimensions did not remain significant unique predictors in the regression model.

Other SDT-related variables were also examined. Subjective Vitality was positively associated with autonomous regulation and autonomy support from nursing-home staff in Kasser and Ryan [42], but not with autonomy support from family or friends. In Solberg et al. [36], exercise was associated with greater increases in autonomous motivation, which in turn were associated with greater increases in Subjective Vitality. By contrast, controlled motivation did not explain changes in Subjective Vitality. In Vermote et al. [39], Subjective Vitality was positively associated with intrinsic goal importance (the degree to which a person assesses a certain goal as important, e.g., growing as a person) and intrinsic goal attainment (the degree of success in pursuing that goal). Older adults who reported higher intrinsic goal attainment and intrinsic goal importance also reported a stronger sense of meaning, which was then associated with higher Subjective Vitality. Only intrinsic goal importance retained a small direct association with Subjective Vitality after accounting for meaning.

Regarding non-SDT antecedents identified by the studies, flow (i.e., state of intrinsic performance with deep immersion) was positively associated with Subjective Vitality in Chang [41], although relatedness satisfaction remained the only significant need-related predictor after flow was entered in the regression model. The number of social contacts, but not frequency of social support, was positively associated with Subjective Vitality in Kasser and Ryan [42]. Ego integrity was positively associated with Subjective Vitality in Van der Kaap-Deeder et al. [38], whereas despair was negatively associated with it. Their mediation model suggested that older adults with higher ego integrity and lower despair reported greater basic psychological need satisfaction, which in turn was associated with higher Subjective Vitality. Finally, meaning in life was positively associated with Subjective Vitality in Vermote et al. [39], and helped explain why intrinsic goal attainment and intrinsic goal importance were associated with higher Subjective Vitality.

#### 3.4.2. Intervention Evidence

Three independent samples assessed intervention effects on Subjective Vitality (Table 4). Chang and Kao [40] found that participants in a leisure education intervention, designed to help them identify and pursue valued leisure activities, reported higher Subjective Vitality than the control group.

Solberg et al. [35,37] examined three exercise programs: endurance, functional, and strength training. All three training conditions showed beneficial short-term effects on Subjective Vitality, but only endurance training showed a clear beneficial effect at the end of the 16-week intervention. At the 1-year follow-up, endurance training showed the most favorable long-term pattern, whereas functional training showed mostly no effects and strength training showed a likely harmful reduction in subjective vitality. Competence satisfaction partly explained some post-test effects, and autonomous motivation appeared relevant for maintaining vitality after the intervention ended. Controlled forms of motivation, namely introjected and external regulation, were associated with short-term gains but did not support long-term maintenance.

Stathi et al. [43] tested ACE (Active, Connected, Engaged), a 6-month peer-volunteering intervention in which trained older volunteers supported inactive and socially disengaged older adults to get out more and engage with local community activities. Subjective Vitality increased in the intervention group and decreased in the control group at 6 months. However, because the confidence interval for the intervention-group change included zero and the study was a feasibility trial, this finding should be interpreted as preliminary evidence of a possible beneficial effect.

### 3.5. Quality Appraisal

In the methodological quality appraisal with the MMAT, three of the eight independent samples were rated as moderate quality (60%), whereas the remaining five were rated as low quality (scores ≤ 40%). All eight studies had clear research questions and collected data appropriate to address them. The most frequent concerns were limited or unclear representativeness of the target population, identified in six independent samples, and incomplete or unclear outcome data, identified in four. Among the three intervention studies, two had incomplete or unclear outcome data, two raised concerns related to the lack of blinding for self-reported vitality outcomes, and two had insufficient reporting of randomization procedures or allocation concealment.

## 4. Discussion

Energy is an important resource for older adults, as it can affect everyday functioning, social participation, and quality of life. Within the SDT framework, Subjective Vitality has been conceptualized as the perceived experience of having energy available to the self and to support self-regulation. However, to date, no scoping review has summarized the literature on Subjective Vitality in older adults. Scoping reviews are particularly useful for mapping the extent and characteristics of a research field, identifying methodological approaches, and highlighting gaps that can guide future research. Thus, the present scoping review aimed to examine the populations and research domains in which Subjective Vitality has been studied, how it has been measured, what psychometric evidence is available, which antecedents, mechanisms, and interventions have been investigated, and what methodological gaps characterize the existing literature.

### 4.1. In Which Population and Research Domains Has Subjective Vitality Been Studied in Older Adults?

A recent review by Logvinov and Loerzel [45] highlighted that the way vitality is expressed and conceptualized may vary across cultural contexts. For example, Dawe et al. [18] found that Italian and USA older adults differed in how they responded to some Subjective Vitality indicators. Specifically, older adults in the USA tended to report higher levels of “liveliness and spark” than their Italian counterparts. These differences may partly reflect cultural representations of aging, such as the tendency in the USA to associate successful aging with activity, youthfulness, energy, and openness to new experiences [46,47,48,49]. In the present scoping review, Subjective Vitality was examined across several countries, including Taiwan, the USA, Italy, Norway, the United Kingdom, Belgium, and France. However, most evidence came from European countries or the USA, with limited representation of non-Western cultural contexts. This lack of cultural diversity may limit our understanding of how Subjective Vitality is experienced, expressed, and measured across older populations, as well as the generalizability of current findings.

Except for Kasser and Ryan’s [42] study, which was conducted with nursing home residents, most independent samples consisted of community-dwelling older-adult populations, without focusing on specific clinical, functional, or social subgroups. Older adults are a highly heterogeneous population, and aging is frequently accompanied by chronic conditions, multimorbidity, and functional limitations [5,6,50]. Therefore, findings based mainly on general or relatively healthy samples may not fully generalize to older adults with poorer health, functional impairment, institutionalization, or greater social vulnerability.

In contrast, Subjective Vitality was examined across heterogeneous research domains, including leisure activities, physical activity, active aging, health, well-being, COVID-19 lockdown, and scale validation. This breadth suggests that Subjective Vitality is relevant to several areas of later life. Leisure and community engagement may become especially salient after retirement, whereas physical activity is central to healthy aging because it contributes to physical and mental health and, among older adults, helps prevent falls and declines in functional ability [51,52]. However, the evidence within each domain remains sparse. Most domains were represented by only one or two independent samples, which limits the ability to identify stable patterns across settings. Thus, the current literature suggests that Subjective Vitality is a flexible construct applicable to several areas of older-adult research, but it has not yet been studied systematically within any specific population or domain.

Collectively, these observations point to a population and context gap in the current evidence base: Subjective Vitality in older adults has been studied predominantly in community-dwelling, relatively healthy, and Western (European or USA) samples, with limited representation of institutionalized older adults, individuals with poorer health or functional impairment, socially vulnerable subgroups, and non-Western cultural contexts. Future research should therefore prioritize these underrepresented populations and settings to clarify how Subjective Vitality is experienced, expressed, and measured across the full diversity of the older adult population.

### 4.2. How Has Subjective Vitality Been Measured, and What Psychometric or Adaptation Evidence Is Available?

Clear definitions and transparent measurement practices are essential for building cumulative evidence and comparing findings across studies [53,54]. Psychometric reporting should also include information on the measurement properties of the instrument, including reliability and validity [55]. In the case of Subjective Vitality, relevant methodological information includes the specific scale version used, number of items, response format, scoring procedure, translation or adaptation procedures, reliability, structural validity, and measurement invariance. The present review showed that two SDT-based instruments were used to assess Subjective Vitality in older adults: the Subjective Vitality Scale (SVS) and the Subjective Vitality/Depletion Scale (SVDS). The SVS was used in most independent samples, which, from its development by Ryan and Frederick [10] to the recent introduction of the SVDS by Frederick and Ryan [19], served as the main instrument for assessing Subjective Vitality within the SDT framework. However, several versions of the SVS were used, with variation in both the number of items and the response scale. This heterogeneity may limit comparability across studies and make it difficult to determine whether differences in findings reflect substantive differences in Subjective Vitality or differences in measurement.

Regarding psychometric and adaptation evidence, one included study was specifically designed as a validation study. Dawe et al. [18] showed that the SVDS had good psychometric properties in an older-adult sample, including evidence of reliability, structural validity, measurement invariance across gender and age groups, partial scalar invariance across countries, and associations with personality, health, and physical activity indicators. Among the SVS-based studies, previous validation evidence in older adults was cited for only two independent samples [41,44]. However, the response formats used in these studies differed from those used in the validation study they cited: 1–7 in Couto et al. [23], 1–5 in Chang [41], and 1–4 in Vanhove-Meriaux et al. [44]. This difference may further limit direct comparability.

Internal consistency was reported for four SVS-based independent samples [35,36,37,41,42,44], but these studies generally did not provide additional evidence on the validity of the specific SVS version used in their own older-adult samples. In addition, two related reports based on the same sample used a single item from the SVS [38,39], for which internal consistency was not applicable and no additional validation evidence was reported. Future studies should therefore report the exact scale version used, justify any item reduction or adaptation, and provide psychometric evidence in older-adult samples whenever possible. In this regard, the SVDS represents the most recent tool, aligned with current SDT conceptualization, and has been validated on a sample of older adults [18]. Overall, although Subjective Vitality was often assessed using established measures, psychometric and adaptation evidence specific to older adults, cultural contexts, and modified scale formats remains limited, representing a methodological gap in the current evidence base.

At the conceptual level, the recently introduced construct of Subjective Depletion was examined in only one included study [18], leaving its conceptualization, distinctiveness from Subjective Vitality, and psychometric properties in older adults largely unexamined. This represents a conceptual gap warranting further theoretical and empirical attention.

### 4.3. Which SDT-Related and Other Antecedents, Mechanisms, and Intervention Approaches Have Been Examined in Relation to Subjective Vitality in Older Adults?

According to SDT, satisfaction of the basic psychological needs for autonomy, competence, and relatedness is a central source of psychological energy and represents a key antecedent of Subjective Vitality [19,20]. Overall, the included studies were consistent with this expectation, as Subjective Vitality was positively associated with autonomy, competence, and relatedness satisfaction. However, the evidence on which specific need was most relevant was inconsistent. Relatedness satisfaction was the only unique predictor in Chang [41] and Vanhove-Meriaux et al. [44], whereas competence satisfaction was the only unique predictor in Van der Kaap-Deeder et al. [38]. Solberg et al. [36] also supported the relevance of competence, although autonomy and relatedness were not included in the same model. Current evidence suggests that need satisfaction is relevant for Subjective Vitality in older adults, but it does not yet allow firm conclusions about the relative importance of autonomy, competence, and relatedness.

These inconsistencies may partly reflect differences in measurement and study context. Chang [41] and Vanhove-Meriaux et al. [44] used five-item versions of the SVS, whereas Van der Kaap-Deeder et al. [38] used a single-item measure. Although single-item measures may reduce participant burden, they may not fully capture the complexity of Subjective Vitality and may influence which predictors emerge as significant. Context may also matter—relatedness may be especially salient in leisure and well-being contexts, whereas competence may become more important when vitality is linked to functioning, coping, or perceived effectiveness in daily life. Future studies should therefore test the three needs simultaneously using validated multi-item measures of Subjective Vitality with consistent response scales, and examine whether the relative importance of autonomy, competence, and relatedness varies across contexts, such as leisure, physical activity, health, or institutional care settings.

Need thwarting was examined only by Vanhove-Meriaux et al. [44]. Although autonomy, competence, and relatedness thwarting were negatively correlated with Subjective Vitality, they did not remain significant unique predictors in the regression model. This finding should be interpreted cautiously because it comes from a single study. However, the recent SDT dual-process model of human energy may help explain this pattern. Frederick and Ryan [19] proposed that Subjective Vitality and Subjective Depletion are distinct but related constructs. From this perspective, need satisfaction may be more closely linked to vitality, whereas need frustration or thwarting may be more closely linked to depletion. Future studies should therefore examine vitality and depletion simultaneously, rather than treating low vitality as equivalent to depletion.

Other SDT-related variables examined in the included studies were autonomy support, autonomous regulation, autonomous motivation, intrinsic goal attainment, and intrinsic goal importance. These findings suggest that more autonomous forms of motivation and support may be relevant for vitality in later life. For example, Kasser and Ryan [42] found that nursing home residents reported higher Subjective Vitality when they experienced greater autonomy support from staff, suggesting that the daily relational environment may be particularly important in institutional settings. Solberg et al. [36] showed that exercise was associated with greater increases in autonomous motivation, which in turn were associated with greater increases in Subjective Vitality. Vermote et al. [39] found that intrinsic goal importance had a small direct association with Subjective Vitality, whereas intrinsic goal attainment was associated with vitality through meaning in life. This suggests that personally meaningful goals may support vitality partly by strengthening the perception that one’s life is meaningful.

Regarding non-SDT variables, the included studies suggest that flow, number of social contacts, ego integrity, and meaning in life may be relevant to Subjective Vitality. Flow and number of social contacts point to the relevance of activity engagement and social embeddedness, two domains that may support vitality by fostering involvement, purpose, and connection. Interestingly, ego integrity and meaning in life both involve coherence and integration of life experiences, although ego integrity refers more specifically to retrospective acceptance of one’s life course, whereas meaning in life also includes present significance and future-oriented purpose. These findings suggest that older adults may experience greater energy and aliveness when they are able to integrate past experiences, accept their life course, and perceive their current life as meaningful.

Lastly, three types of interventions were examined in relation to Subjective Vitality: leisure education, physical activity programs, and peer-supported active aging programs. Overall, the intervention evidence suggests that Subjective Vitality may be improved through activity-based or engagement-oriented programs, although findings remain limited. In the Solberg project, endurance training showed the clearest and most sustained pattern, whereas functional and strength training showed less stable effects over time.

Across both SDT and non-SDT antecedents and intervention types, most variables and mechanisms were examined in only one or two independent samples. This sparse and fragmented evidence base represents a methodological gap. Currently, this scenario precludes firm conclusions about which antecedents and mechanisms are most important for Subjective Vitality in older adults, and about the relative efficacy of different intervention types.

### 4.4. What Methodological Gaps Characterize the Current Literature?

Some of the limitations highlighted in the previous paragraphs were also reflected in the quality appraisal. Several recurrent methodological limitations were identified. Representativeness was limited in three independent samples and unclear in three others, mainly because of convenience recruitment, selective online samples, or insufficient reporting of the sampling frame and recruitment procedures, which made it difficult to judge whether the samples adequately reflected the target older-adult population. Although most studies used theoretically appropriate measures, some relied on abbreviated scales or single-item vitality measures, with limited psychometric information specific to older adults. In addition, most observational studies were cross-sectional, limiting conclusions about directionality and causal inference. Among intervention studies, the main concerns were incomplete outcome data, the difficulty of blinding participants or assessors when using self-reported vitality outcomes, and insufficient reporting of randomization procedures or allocation concealment. Overall, these findings point to the need for methodologically stronger studies, especially longitudinal and intervention studies, using clearer sampling procedures, validated measures, adequate reporting, and designs capable of testing temporal and causal relationships.

### 4.5. Limitations

Although this scoping review is, to our knowledge, the first to map Subjective Vitality among older adults from an SDT-based perspective, it has some limitations. First, we did not search for gray literature, which may have led to the omission of relevant unpublished evidence. Second, we defined older adults as individuals aged 65 years or older, following a more recent and commonly used threshold in aging research. However, earlier studies often considered individuals aged 60 years and older as older adults. Therefore, our eligibility criterion may have led to the exclusion of older studies that adopted a broader age threshold, or samples including a small proportion of participants below 65 years. Although this choice increased the conceptual consistency of the review, it may have reduced the coverage of earlier research on Subjective Vitality in later life.

## 5. Conclusions

This scoping review identified and mapped research on Subjective Vitality among older adults from an SDT perspective. The results showed that most studies were conducted in Western countries, mainly Europe and the USA, indicating limited cultural diversity in the existing evidence base. Moreover, research was mainly conducted with broadly defined samples of older adults, with limited attention to specific clinical, functional, institutionalized, or socially vulnerable subgroups. Although Subjective Vitality was examined across several research domains, including leisure, physical activity, health, well-being, active aging, COVID-19 lockdown, and scale validation, evidence within each domain remains limited.

Nearly all studies used the Subjective Vitality Scale (SVS), which may facilitate some comparison across studies. However, comparability remains limited because different versions of the SVS were used, response formats varied, and information on validation, adaptation, and psychometric properties was often incomplete. The recent validation of the Subjective Vitality/Depletion Scale (SVDS) is a notable addition, but further evidence is needed across different populations, languages, and cultural contexts.

This scoping review mapped the antecedents and mechanisms of Subjective Vitality, including basic psychological need satisfaction, autonomy support, autonomous regulation, intrinsic goal attainment and importance, flow, number of social contacts, ego integrity, and meaning in life. Intervention studies examining leisure education and endurance reported some association with Subjective Vitality in older adults. However, findings were limited and sometimes inconsistent, particularly regarding the relative importance of autonomy, competence, and relatedness satisfaction.

Overall, the present review shows that research on Subjective Vitality in older adults is promising but still fragmented. Future studies should use clearer sampling procedures, validated and well-reported measures, and stronger longitudinal and intervention designs capable of testing temporal and causal relationships. Given that basic psychological needs represent the principal theoretical antecedents of Subjective Vitality within SDT, future research should also examine the individual and combined roles of autonomy, competence, and relatedness across different older-adult populations and research domains. Finally, this review focused primarily on Subjective Vitality. However, recent developments in SDT propose a dual-process model in which subjective vitality and depletion are distinct but related dimensions of perceived energy. Because only one included study considered subjective depletion, future research should examine both constructs in older adults.

## Figures and Tables

**Table 1:** Characteristics of included reports.

Reference	Year	Sample Characteristics				Context	Study Design
		N (F%)	Mean (SD)	Country	Population		
Chang & Kao [40]	2019	42 (n.r.)	69.12 (4.09)	Taiwan	Not enough information was provided	Leisure activities	NRSI
Chang [41]	2020	257 (63.4%)	71 (8.77)	Taiwan	General (community-dwelling)	Leisure activities	Cross-sectional
Kasser & Ryan [42]	1999	50 (78%)	83 (n.r.)	USA	Nursing home residents	Health	Cross-sectional
Dawe et al. [18]	2026	726 (51.1%)	72.57 (6.49)	Italy and USA	General (Setting not specified) ^a^	General validation	Cross-sectional
Solberg et al. * [35]	2012	138 (68%)	74.2 (4.5)	Norway	General (community-dwelling)	Physical Activity	RCT
Solberg et al. * [36]	2013	118 (68%)	74.3 (4.6)	Norway	General (community-dwelling)	Physical Activity	RCT
Solberg et al. * [37]	2014	62 (61.29%)	74.48 (5.12)	Norway	General (community-dwelling)	Physical Activity	RCT
Stathi et al. [43]	2020	54 (55.55%)	72.39 (6.69)	UK	General (community-dwelling)	Active aging	RCT
Van der Kaap-Deeder et al. * [38]	2022	693 (62.1%)	70.06 (4.48)	Belgium	General (community-dwelling)	COVID-19 lockdown	Cross-sectional
Vanhove-Meriaux et al. [44]	2018	182 (56.04%)	73.33 (7.07)	France	General (community-dwelling)	Well-being	Cross-sectional
Vermote et al. * [39]	2023	693 (62.1%)	70.06 (4.48)	Belgium	General (community-dwelling)	COVID-19 lockdown	Prospective

Note. n.r. = not reported; RCT = randomized controlled trial; NRSI = non-randomized studies with intervention. * The tables report all included reports. However, numerical summaries in the text are based on independent samples. The three Solberg reports [35,36,37] were treated as one exercise-intervention project. Van der Kaap-Deeder et al. [38] and Vermote et al. [39] were treated as one COVID-19 survey project because they used the same sample. ^a^ Living setting not explicitly stated; sample recruited online from independent community members and screened to exclude medical and psychological conditions.

**Table 2:** Measures and psychometric evidence for Subjective Vitality.

Reference	Measure	N Items	Score Range	Language	Psychometric Properties
Chang & Kao [40]	SVS	7	1–5	Chinese/Taiwanese Mandarin, inferred; language not explicitly reported.	Previous validation cited; no older-adult-specific validation/adaptation evidence reported in the study.
Chang [41]	SVS	5	1–5	Chinese/Taiwanese Mandarin, inferred; language not explicitly reported.	Previous validation in older adults cited. Reliability (α) = 0.87
Kasser & Ryan [42]	SVS	4	1–7	English	Previous validation cited; no older-adult-specific validation/adaptation evidence reported in the study. Reliability (α) = 0.82
Dawe et al. [18]	SVDS	3	1–7	Italian and English	Structural validity, measurement invariance, reliability, and convergent/nomological validity were examined using CFA, MG-CFA, and correlations. Reliability = 0.904 (alpha), 0.883 (CR);
Solberg et al.* [35,36,37]	SVS	6	1–7	Norwegian	Previous validation cited; no older-adult-specific validation/adaptation evidence reported in the study. Reliability (α) = 0.91
Stathi et al. [43]	SVS	6	1–7	English	Previous validation cited; no older-adult-specific validation/adaptation evidence reported in the study. Reliability not reported.
Van der Kaap-Deeder et al. and Vermote et al. * [38,39]	SVS	1	1–4	Dutch	Single-item assessment; reliability not applicable and validation evidence not reported.
Vanhove-Meriaux et al. [44]	SVS	5	1–4	French	Previous validation in older adults cited. Reliability (α) = 0.72

Note. SVS = Subjective Vitality Scale; SVDS = Subjective Vitality/Depletion Scale; CR = composite reliability; CFA = confirmatory factor analysis; MG-CFA = multigroup confirmatory factor analysis. * The tables report all included reports. However, numerical summaries in the text are based on independent samples. The three Solberg reports [35,36,37] were treated as one exercise-intervention project. Van der Kaap-Deeder et al. [38] and Vermote et al. [39] were treated as one COVID-19 survey project because they used the same sample.

**Table 3:** Evidence on antecedents and mechanisms of Subjective Vitality.

Reference	Theoretical SDT Antecedents	Non-SDT Antecedents	Synthesis of Results
Chang [41]	Autonomy, competence, and relatedness satisfaction	Flow	Subjective vitality showed a significant positive correlation with Autonomy, Competence, Relatedness, and Flow. The regression model showed that autonomy, competence, and relatedness significantly predicted subjective vitality. However, after flow was entered in the model, only relatedness predicted subjective vitality.
Kasser & Ryan [42]	Autonomous regulation, autonomy support from staff, autonomy support from family/friends, and quality of relatedness with family/friends	Frequency of social support, number of social contacts	Subjective Vitality was positively associated with autonomous regulation, autonomy support from staff, and number of social contacts. Associations with family/friend autonomy support, quality of relatedness, and frequency of social support were not significant.
Solberg et al. * [36]	Competence satisfaction, autonomous motivation, and controlled motivation	Physical activity	Changes in autonomous motivation and perceived competence were positively associated with changes in vitality. The indirect exercise–vitality pathway through autonomous motivation was supported, whereas the pathway through perceived competence was only marginal. The indirect path from impersonal orientation through controlled motivation was not supported, and causality orientations did not significantly moderate the effects of exercise on motivational variables.
Van der Kaap-Deeder et al. * [38]	Basic Psychological Need satisfaction, autonomy, competence, and relatedness satisfaction	Ego integrity, Despair	Subjective Vitality showed a positive correlation with Ego integrity, Basic Psychological Need satisfaction, and a negative correlation with Despair. The mediation model showed that Basic Psychological Need satisfaction mediated the relationship of Ego integrity and Despair with Subjective Vitality. In supplementary analyses separating the three needs, competence satisfaction was the only need uniquely associated with vitality; autonomy and relatedness satisfaction did not uniquely predict vitality.
Vanhove-Meriaux et al. [44]	Autonomy, competence, and relatedness satisfaction, autonomy, competence, and relatedness thwarting	n.a.	Subjective Vitality was positively associated with autonomy, competence, and relatedness satisfaction, and negatively associated with the frustration of the three needs. However, in the regression model, only relatedness satisfaction uniquely predicted vitality; autonomy satisfaction, competence satisfaction, and all need-thwarting dimensions were not significant unique predictors.
Vermote et al. * [39]	Intrinsic goal attainment, intrinsic goal importance	Meaning	Subjective Vitality showed a positive correlation with Intrinsic goal attainment (T2), intrinsic goal importance (T2), and meaning. Intrinsic goal attainment and importance were indirectly associated with vitality through meaning. Intrinsic goal importance also had a small direct effect.

Note. n.a. = not applicable. * The tables report all included reports. However, numerical summaries in the text are based on independent samples. The three Solberg reports [35,36,37] were treated as one exercise-intervention project. Van der Kaap-Deeder et al. [38] and Vermote et al. [39] were treated as one COVID-19 survey project because they used the same sample.

**Table 4:** Intervention studies assessing changes in Subjective Vitality.

Reference	Intervention	Control	Duration	Synthesis of Results
Chang & Kao [40]	Leisure education program (the focus is to identify and help them pursue valued leisure activities).	No program	12 weeks	Participants in the leisure education group showed higher post-intervention Subjective Vitality than controls.
Solberg et al. [35]	Three intervention groups: endurance, functional, and strength training programs.	Waiting list control	16 weeks.	All three training programs showed short-term benefits, but only endurance training showed a clear beneficial effect at 16 weeks. Competence satisfaction partly explained some post-test effects, and autonomy support moderated selected effects.
Solberg et al. [37]	Three intervention groups: endurance, functional, and strength training programs.	No control group at follow-up; wait-list controls were excluded	1-year follow-up after the 4-month intervention.	At 1-year follow-up, endurance training showed the most favorable pattern, functional training showed trivial effects, and strength training showed a likely harmful reduction. Autonomous motivation supported maintenance of vitality, whereas introjected and external regulation showed only short-term associations.
Stathi et al. [43]	ACE (Active, Connected, Engaged) program, a peer-volunteering intervention in which trained older volunteers supported inactive and socially disengaged older adults to get out more and engage with local community activities.	Written materials about local initiatives only	6 months.	The ACE intervention group showed an increase in Subjective Vitality from baseline to 6 months, whereas the control group showed a decrease. However, as this was a feasibility trial and the intervention-group confidence interval included zero, the finding should be interpreted as preliminary evidence of a possible positive effect on vitality.

Note. n.a. = not applicable.

## Data Availability

No new data were created or analyzed in this study. Data sharing is not applicable to this article.

## Supplementary Materials

The following supporting information can be downloaded at: https://www.mdpi.com/article/10.3390/geriatrics11040091/s1, File S1: Preferred Reporting Items for Systematic reviews and Meta-Analyses extension for Scoping Reviews (PRISMA-ScR) Checklist. File S2: search string.

## References

[1] United Nations (2023). World Social Report 2023: Leaving No One Behind in an Ageing World.

[2] United Nations (2024). World Population Ageing 2023: Challenges and Opportunities of Population Ageing in the Least Developed Countries.

[3] Grünwald O., Damman M., Henkens K. (2021). The Differential Impact of Retirement on Informal Caregiving, Volunteering, and Grandparenting: Results of a 3-Year Panel Study. J. Gerontol. Ser. B.

[4] Kalbarczyk M., Łopaciuk-Gonczaryk B. (2022). Social and Private Activity after Retirement-Substitutes or Complements. BMC Geriatr..

[5] Jindai K., Nielson C.M., Vorderstrasse B.A., Quiñones A.R. (2016). Multimorbidity and Functional Limitations Among Adults 65 or Older, NHANES 2005–2012. Prev. Chronic Dis..

[6] Gianfredi V., Nucci D., Pennisi F., Maggi S., Veronese N., Soysal P. (2025). Aging, Longevity, and Healthy Aging: The Public Health Approach. Aging Clin. Exp. Res..

[7] Avlund K. (2010). Fatigue in Older Adults: An Early Indicator of the Aging Process?. Aging Clin. Exp. Res..

[8] Singh S., Sharma A., Rani R. (2023). Effect of Age and Gender on Subjective Vitality of Adults. IAHRW Int. J. Soc. Sci. Rev..

[9] Ehrenkranz R., Rosso A.L., Sprague B.N., Tian Q., Gmelin T., Bohnen N., Simonsick E.M., Glynn N.W., Rosano C. (2021). Functional Correlates of Self-Reported Energy Levels in the Health, Aging and Body Composition Study. Aging Clin. Exp. Res..

[10] Ryan R.M., Frederick C. (1997). On Energy, Personality, and Health: Subjective Vitality as a Dynamic Reflection of Well-Being. J. Personal..

[11] Valentine R.J., Woods J.A., McAuley E., Dantzer R., Evans E.M. (2011). The Associations of Adiposity, Physical Activity and Inflammation with Fatigue in Older Adults. Brain Behav. Immun..

[12] Hardy S.E., Studenski S.A. (2008). Fatigue and Function Over 3 Years Among Older Adults. J. Gerontol. Ser. A Biol. Sci. Med. Sci..

[13] Barros D., Borges-Machado F., Silva-Fernandes A., Ribeiro O., Carvalho J. (2024). Do Physical Fitness and Cognitive Function Mediate the Relationship between Basic Activities of Daily Living and Quality of Life in Older Adults with Dementia?. Qual. Life Res..

[14] Feng C., Shi Z., Tian Y., Ma C., Liu J. (2024). A Study of the Relationship between Leisure-Time Physical Activity and Residents’ Quality of Life: Chain Mediation of Sleep Quality and Depression. Curr. Psychol..

[15] Geigl C., Loss J., Leitzmann M., Janssen C. (2023). Social Factors of Health-Related Quality of Life in Older Adults: A Multivariable Analysis. Qual. Life Res..

[16] Van Roekel E.H., Duchâteau J., Bours M.J.L., Van Delden L., Breedveld-Peters J.J.L., Koole J.L., Kenkhuis M., Van Den Brandt P.A., Jansen R.L., Kant I. (2020). Longitudinal Associations of Light-Intensity Physical Activity with Quality of Life, Functioning and Fatigue after Colorectal Cancer. Qual. Life Res..

[17] Chirico A., Dawe J., Alivernini F., Manganelli S., Baiocco R., Zacchilli M., Cazzolli B., Raimondi G., Palombi T., Lucidi F. (2025). Does Using Technological Devices Motivate Older Adults to Engage in Physical Activities? A Systematic Review and Meta-Analysis. Topoi.

[18] Dawe J., Cavicchiolo E., Palombi T., Frederick C.M., Chirico A., Lucidi F., Alivernini F. (2026). Measuring Subjective Vitality and Depletion in Older People from a Self-Determination Theory Perspective: A Dual Country Study. Exp. Aging Res..

[19] Frederick C., Ryan R.M., Ryan R.M. (2023). The Energy behind Human Flourishing: Theory and Research on Subjective Vitality. The Oxford Handbook of Self-Determination Theory.

[20] Ryan R.M., Deci E.L. (2008). From Ego Depletion to Vitality: Theory and Findings Concerning the Facilitation of Energy Available to the Self. Soc. Personal. Psychol. Compass.

[21] Adie J.W., Duda J.L., Ntoumanis N. (2008). Autonomy Support, Basic Need Satisfaction and the Optimal Functioning of Adult Male and Female Sport Participants: A Test of Basic Needs Theory. Motiv. Emot..

[22] Raimondi G., Zacchilli M., Frederick C.M., Alivernini F., Manganelli S., Cavicchiolo E., Lucidi F., Palombi T., Chirico A., Dawe J. (2025). Measuring Vitality and Depletion During Adolescence: Validation of the Subjective Vitality/Subjective Depletion Scale in a Sample of Italian Students. Pediatr. Rep..

[23] Couto N., Antunes R., Monteiro D., Moutão J., Marinho D., Cid L. (2017). Validação Da Subjective Vitality Scale e Estudo Da Vitalidade Nos Idosos Em Função Da Sua Atividade Física. Rev. Bras. Cineantropometria Desempenho Hum..

[24] Bostic T.J., Rubio D.M., Hood M. (2000). A Validation of the Subjective Vitality Scale Using Structural Equation Modeling. Soc. Indic. Res..

[25] Kawabata M., Yamazaki F., Guo D.W., Chatzisarantis N.L.D. (2017). Advancement of the Subjective Vitality Scale: Examination of Alternative Measurement Models for Japanese and Singaporeans. Scand. J. Med. Sci. Sports.

[26] Liu J.-D., Chung P.-K. (2019). Factor Structure and Measurement Invariance of the Subjective Vitality Scale: Evidence from Chinese Adolescents in Hong Kong. Qual. Life Res..

[27] Peters M.D.J., Godfrey C.M., Khalil H., McInerney P., Parker D., Soares C.B. (2015). Guidance for Conducting Systematic Scoping Reviews. Int. J. Evid.-Based Healthc..

[28] Munn Z., Peters M.D.J., Stern C., Tufanaru C., McArthur A., Aromataris E. (2018). Systematic Review or Scoping Review? Guidance for Authors When Choosing between a Systematic or Scoping Review Approach. BMC Med. Res. Methodol..

[29] Peters M.D.J., Godfrey C., McInerney P., Khalil H., Larsen P., Marnie C., Pollock D., Tricco A.C., Munn Z. (2022). Best Practice Guidance and Reporting Items for the Development of Scoping Review Protocols. JBI Evid. Synth..

[30] Tricco A.C., Lillie E., Zarin W., O’Brien K.K., Colquhoun H., Levac D., Moher D., Peters M.D.J., Horsley T., Weeks L. (2018). PRISMA Extension for Scoping Reviews (PRISMA-ScR): Checklist and Explanation. Ann. Intern. Med..

[31] United Nations International Day of Older Persons. https://www.un.org/en/observances/older-persons-day.

[32] Ouzzani M., Hammady H., Fedorowicz Z., Elmagarmid A. (2016). Rayyan-a Web and Mobile App for Systematic Reviews. Syst. Rev..

[33] Hong Q.N., Fàbregues S., Bartlett G., Boardman F., Cargo M., Dagenais P., Gagnon M.-P., Griffiths F., Nicolau B., O’Cathain A. (2018). The Mixed Methods Appraisal Tool (MMAT) Version 2018 for Information Professionals and Researchers. Educ. Inf..

[34] Crossman S., Drummond M., Elliott S., Kay J., Montero A., Petersen J.M. (2024). Facilitators and Constraints to Adult Sports Participation: A Systematic Review. Psychol. Sport Exerc..

[35] Solberg P.A., Hopkins W.G., Ommundsen Y., Halvari H. (2012). Effects of Three Training Types on Vitality among Older Adults: A Self-Determination Theory Perspective. Psychol. Sport Exerc..

[36] Solberg P.A., Halvari H., Ommundsen Y. (2013). Linking Exercise and Causality Orientations to Change in Well-being among Older Adults: Does Change in Motivational Variables Play a Role?. J. Appl. Soc. Psychol..

[37] Solberg P.A., Halvari H., Ommundsen Y., Hopkins W.G. (2014). A 1-Year Follow-Up on Effects of Exercise Programs on Well-Being in Older Adults. J. Aging Phys. Act..

[38] Van Der Kaap-Deeder J., Vermote B., Waterschoot J., Soenens B., Morbée S., Vansteenkiste M. (2022). The Role of Ego Integrity and Despair in Older Adults’ Well-Being during the COVID-19 Crisis: The Mediating Role of Need-Based Experiences. Eur. J. Ageing.

[39] Vermote B., Morbée S., Soenens B., Vansteenkiste M., Waterschoot J., Beyers W., Van Der Kaap-Deeder J. (2023). How Do Late Adults Experience Meaning During the COVID-19 Lockdown? The Role of Intrinsic Goals. J. Happiness Stud..

[40] Chang L.-C., Kao I.-C. (2019). Enhancing Social Support and Subjective Vitality among Older Adults through Leisure Education. Int. Psychogeriatr..

[41] Chang L.-C. (2020). Relationship between Flow Experience and Subjective Vitality among Older Adults Attending Senior Centres. Leis. Stud..

[42] Kasser V.G., Ryan R.M. (1999). The Relation of Psychological Needs for Autonomy and Relatedness to Vitality, Well-Being, and Mortality in a Nursing Home. J. Appl. Soc. Psychol..

[43] Stathi A., Withall J., Thompson J.L., Davis M.G., Gray S., De Koning J., Parkhurst G., Lloyd L., Greaves C., Laventure R. (2020). Feasibility Trial Evaluation of a Peer Volunteering Active Aging Intervention: ACE (Active, Connected, Engaged). Gerontologist.

[44] Vanhove-Meriaux C., Martinent G., Ferrand C. (2018). Profiles of Needs Satisfaction and Thwarting in Older People Living at Home: R Elationships with Well-being and Ill-being Indicators. Geriatr. Gerontol. Int..

[45] Logvinov I.I., Loerzel V. (2026). Vitality in Older Adults: A State-of-the-Science Review. Arch. Gerontol. Geriatr..

[46] Karlin N.J., Weil J., Saratapun N., Pupanead S., Kgosidialwa K. (2014). Etic and Emic Perspectives on Aging Across Four Countries: Italy, Thailand, Botswana, and the United States. Ageing Int..

[47] Reich A.J., Claunch K.D., Verdeja M.A., Dungan M.T., Anderson S., Clayton C.K., Goates M.C., Thacker E.L. (2020). What Does “Successful Aging” Mean to You?—Systematic Review and Cross-Cultural Comparison of Lay Perspectives of Older Adults in 13 Countries, 2010–2020. J. Cross-Cult. Gerontol..

[48] Schiffman L.G., Sherman E. (1991). Value Orientations of New-Age Elderly: The Coming of an Ageless Market. J. Bus. Res..

[49] Westerhof G.J., Whitbourne S.K., Freeman G.P. (2012). The Aging Self in a Cultural Context: The Relation of Conceptions of Aging to Identity Processes and Self-Esteem in the United States and the Netherlands. J. Gerontol. Ser. B.

[50] Yang Y., Lee L.C. (2010). Dynamics and Heterogeneity in the Process of Human Frailty and Aging: Evidence from the U.S. Older Adult Population. J. Gerontol. Ser. B Psychol. Sci. Soc. Sci..

[51] Bull F.C., Al-Ansari S.S., Biddle S., Borodulin K., Buman M.P., Cardon G., Carty C., Chaput J.-P., Chastin S., Chou R. (2020). World Health Organization 2020 Guidelines on Physical Activity and Sedentary Behaviour. Br. J. Sports Med..

[52] World Health Organization Ageing and Health. https://www.who.int/news-room/fact-sheets/detail/ageing-and-health.

[53] Dodge R., Daly A.P., Huyton J., Sanders L.D. (2012). The Challenge of Defining Wellbeing. Int. J. Wellbeing.

[54] Dolan P., Metcalfe R. (2012). Measuring Subjective Wellbeing: Recommendations on Measures for Use by National Governments. J. Soc. Policy.

[55] Gagnier J.J., Lai J., Mokkink L.B., Terwee C.B. (2021). COSMIN Reporting Guideline for Studies on Measurement Properties of Patient-Reported Outcome Measures. Qual. Life Res..

