# Supplementary material for: The Role of Subjective Vitality Among Older Adults from a Self-Determination Theory Perspective: A Scoping Review
Source: Geriatrics (Basel). 2026 Jul 20;11(4):91. doi: 10.3390/geriatrics11040091 (PMC13397999; doi:10.3390/geriatrics11040091)
Supplement: Supplementary file 1 [file geriatrics-11-00091-s001.zip › File S2 Search String.pdf]

("≥65 years" OR "65 and older" OR "65 or older" OR "65 years" OR "65+ years" OR "age: > 65" OR "aging adults" OR "elder*" OR "eldest" OR "older adult*" OR "older age" OR "older population" OR "older people" OR "older person*" OR "over 65")

AND

("vitality" OR "vital*" OR "subjective vitality" OR "energy" OR “Alive*”)

NOT

("self determin*" OR "self-determin*"OR "SDT" OR "autonomous motivation" OR "autonomous regulation" OR "intrinsic motivation" OR "intrinsic regulation" OR "intrinsic interest" OR "identified motivation" OR "identified regulation" OR "introjected motivation" OR "introjected regulation" OR "controlled motivation" OR "controlled regulation" OR "extrinsic motivation" OR "extrinsic regulation" OR "external motivation" OR "external regulation" OR "amotivation" OR "basic psychological needs" OR "psychological need" OR "Psychological need satisfaction" OR "BPN" OR "basic needs" OR “basic need” OR "need fulfil*" OR "need satisf*" OR "need support*" OR "need frustrat*" OR "need thwart*" OR "autonomy" OR "competence" OR "relatedness")
